# Effects of *In Vitro* Digestion on the Antioxidant Activity of Three Phenolic Extracts from Olive Mill Wastewaters

**DOI:** 10.3390/antiox12010022

**Published:** 2022-12-22

**Authors:** Dario Mercatante, Diana Ansorena, Agnese Taticchi, Iciar Astiasarán, Maurizio Servili, Maria Teresa Rodriguez-Estrada

**Affiliations:** 1 Department of Agricultural and Food Sciences, Alma Mater Studiorum—University of Bologna, 40127 Bologna, Italy; 2 Department of Nutrition, Food Science and Physiology, School of Pharmacy and Nutrition, Universidad de Navarra, 31008 Pamplona, Spain; 3 Department of Agricultural, Food and Environmental Sciences, University of Perugia, 06126 Perugia, Italy; 4 Interdepartmental Centre for Industrial Agrofood Research, Alma Mater Studiorum—University of Bologna, 47521 Cesena, Italy

**Keywords:** phenolic extract, olive by-products, *in vitro* digestion, antioxidant activity, 2,2-diphenyl-1-picrylhydrazyl (DPPH^•^), 2,2-azinobis-(3-ethylbenzothiazoline-6-sulfonate) (ABTS^•+^), oxygen radical absorbance capacity (ORAC), high-performance liquid chromatography (HPLC)

## Abstract

The aim of this study was to assess the impact of *in vitro* digestion on the antioxidant activity of three extracts rich in phenols (two purified organic extracts (A20, A21) and one powdered extract stabilized with maltodextrins (SP)) obtained from olive mill wastewaters (OMWW). The content and composition of phenols and antioxidant activity was determined before and after *in vitro* digestion. The phenol content of the A20 and A21 samples were higher (>75%) than that of the SP sample before *in vitro* digestion. After the entire *in vitro* digestion, 89.3, 76.9, and 50% loss of phenols was found in A20, A21 and SP, respectively. ABTS^•+^ and ORAC values decreased during *in vitro* digestion of A20 and A21 samples, while they remained almost constant in SP. IC_50_ increased during digestion of A20 and A21, evidencing a loss of antioxidant capacity after the intestinal phase; an opposite IC_50_ trend was noted in SP, confirming the protective role of maltodextrins. For these reasons, SP represents a promising formulation to be used in the food field.

## 1. Introduction

Oxidation is one of the main degradation reactions that occurs in living organisms, involving numerous enzymatic systems. Molecular oxygen (O_2_) and its extremely reactive derivatives (reactive oxygen species, ROS) can induce cellular damage, mainly by targeting polyunsaturated fatty acids in the cell membrane and DNA [1]. Nevertheless, organisms count on several antioxidant defense mechanisms to mitigate such damages. The effectiveness of these mechanisms tends to decline with aging [2], so ROS can accumulate and thus promote the onset of chronic and neurodegenerative diseases, including diabetes and Alzheimer’s [3,4].

One of the most relevant areas of current research in nutrition and food formulation is the search for compounds that can reduce the production and/or reactivity of ROS. This trend, together with the need for improving the sustainability of food production, has directed both industry and basic research towards the search for natural, traditional products (or their extracts) with a high antioxidant activity [5,6]. Natural extracts or essential oils derived from agri-food waste and by-products could be useful to replace synthetic food antioxidants in food product formulations, due to their high content of bioactive compounds (i.e., phenolic compounds, carotenoids or terpenes) that possess antioxidant and antimicrobial effects [7,8].

In this context, olive mill wastewater (OMWW), which is generated in large amounts during olive processing to produce virgin olive oils, represents one of the most interesting and abundant food by-products. The annual world production of wastewater is estimated to be between 7 and 30 million m^3^ [9]. OMWW composition varies from both qualitative and quantitative standpoints depending on the olive cultivar, pedo-climatic conditions, cultivation practices and the oil separation technique. OMWW has a high content of organic compounds (sugars, tannins, pectins and phenolic substances) and mineral salts [10]; in particular, phenolic compounds are one of the main components, varying from 0.6 to 2.0% [9]. Due to its organic composition and high generation rate, OMWW is indeed a highly polluting by-product; the significant amounts of polyphenols contained therein might have negative effects on ecosystems due to their antibacterial, phytotoxic and antinutritional properties, as well as due to their resistance to degradation [10].

However, OMWW could be a source of hydrophilic phenols (mostly secoiridoid derivatives, which are only found in the *Oleaceae* family) with antioxidant, antibacterial and anti-inflammatory activity [11,12]. The phenolic fraction can be extracted from OMWW and purified using diverse methods, such as membrane filtration. Ianni et al. [13] demonstrated that a three-step membrane filtration (microfiltration + ultrafiltration + reverse osmosis) is a good technological procedure to extract and purify OMWW phenols, which can be used for further applications in the food, pharmaceutical or cosmetic fields [12,14,15].

While the phenolic profile of extracts obtained from OMWW has been abundantly characterized, there are no studies available in the literature regarding the *in vitro* digestion of pure extracts and their bioavailability, as reported by Reboredo-Rodríguez et al. [16]. In fact, most scientific investigations have focused on the study of the bioaccessibility of the single phenolic compounds, such as hydroxytyrosol (3,4-DHPEA) and verbascoside, in the form of pure standards or extracted from by-products (i.e., OMWW, olive pomace and leaves) [16]; however, to the best of our knowledge, no study has examined the bioaccessibility of phenolic extracts derived from OMWW containing more than one phenolic compound. In this work, three extracts rich in phenols obtained from the purification of OMWW were subjected to *in vitro* digestion and characterized. The content and composition of phenols and antioxidant activity was determined before and after *in vitro* digestion.

## 2. Materials and Methods

### 2.1. Phenolic Extract

Three samples of phenolic extracts (spray-dried (SP), A20 and A21) obtained from the purification of OMWW were supplied by the Department of Agricultural, Food and Environmental Sciences of the University of Perugia (Perugia, Italy).

To produce A20 and A21 phenolic extracts, fresh OMWWs from a mixture of olive cultivars (harvested in the Umbria region, Italy) were obtained from different harvest years (2020, with prevalence of Coratina cv.; 2021 with prevalence of Frantoio, Moraiolo and Dolce Agogia cvs.); the OMWWs were subjected to a 12 h enzymatic hydrolysis at 20 °C and then filtered by a 3-step membrane system (microfiltration + ultrafiltration + reverse osmosis) [13]. A crude phenolic concentrate (CPC) was thus obtained, which was further treated as described by Menchetti et al. [17]: 100 mL of CPC were homogenized with 50 mL of ethyl acetate for 3 min and the organic phase was recovered; this process was repeated twice. Anhydrous sodium was then added to the entire mixture, which was subsequently filtered with a fluted filter to remove residual water. Ethyl acetate was removed with a rotavapor at 35 °C; the extract was then redissolved in 5 mL of ethanol, which was evaporated under nitrogen flow and stored at −20 °C.

For the preparation of the SP extract, olives from Moraiolo cultivar that were harvested in Umbria (Central Italy) were used to obtain fresh OMWWs and the corresponding CPC as described above. The CPC then had maltodextrin (1:1, d.w.) added as a support, was spray-dried to obtain a powder formulation and stored at room temperature [18].

### 2.2. In Vitro Digestion

The three extracts were subjected to an *in vitro* digestion process using the procedure described by Gayoso et al. (2018) [19], modified following the Infogest method [20]. Briefly, the samples (200 mg for A20 and A21, and 500 mg for SP) were placed in 3 Falcon tubes, dissolved in distilled water and heated in a water bath at 37 °C under stirring. After pH adjustment to 6.5 with 1 M sodium bicarbonate, oral digestion was initiated by adding an α-amylase solution. Afterwards, the pH was again adjusted to 2.5 using 3 M HCl, and 1 tube was immediately frozen (−20 °C). To simulate gastric digestion, pepsin was then added to the 2 remaining tubes; after the set reaction time (2 h), pH of 6.5 was obtained using 1 M sodium bicarbonate and another tube was immediately frozen. The last digestion phase (intestinal) was launched by adding a mixture of pancreatin solution and bile extract (1:1, *v*/*v*); after 2 h, pH was adjusted to 7.5 using 1 M sodium bicarbonate and the last tube was placed in the freezer. Table 1 reports the composition of the simulating digestive fluids.

All digestive tubes were maintained at −20 °C for no longer than 2 days. The 3 tubes were thawed (4 °C), then centrifuged at 4000× *g* (A-4-62 Rotor, Model 5810R centrifuge, Eppendorf, Barcelona, Spain) for 40 min at 4 °C, and the supernatant was collected and lyophilized for further analysis. The supernatant from the first tube represents the bioaccessible fraction from oral digestion (OD), whereas those from the second and third tubes represent the bioaccessible fraction from gastric digestion (OD + GD) and intestinal digestion (ID + OD + GD), respectively. For each type of extract, three independent triplicates of the digestion process were run.

### 2.3. Total Polyphenol (TP) Content

The TP content was measured by the Folin–Ciocalteu assay [21]. Briefly, 15 μL of sample were mixed with distilled water and the Folin–Ciocalteu reagent. After 2 min, sodium carbonate was added and, after 2 h, the absorbance was measured at 765 nm using a microplate reader (FLUOstar Omega, BMG Labtech, Ortenberg, Germany). Gallic acid (GA) was used to build the calibration curve (concentration range: 5–2000 µg/mL) and the results were expressed as mg GA equivalents (GAE)/100 g dry weight (d.w.) phenolic extract.

### 2.4. 2,2-Diphenyl-1-Picrylhydrazyl (DPPH^•^) Inhibition

Antioxidant activity was monitored using the scavenging effect of radicals on DPPH^•^ (Sigma-Aldrich Co., St. Louis, MO, USA) [22]. Briefly, samples’ stock solution (A20 and A21 = 25 µg/mL of methanol; SP = 2.5 mg/mL of water) were diluted, so as to obtain 10 concentration levels for each solution; 150 µL of each diluted solution were mixed with 150 µL of DPPH^•^ solution (0.04 mg/mL). The progress of the reaction was checked every 15 min, for a total of 90 min, by using a spectrophotometer (UV PowerWave XS, BioTek Instruments, Inc., Winooski, VT, USA) at an absorbance wavelength of 517 nm. To determine the radical scavenging activity (% of inhibition), the following formula was utilized:

Inhibition (%) = 1 − (Abs_sample_ − Abs_blank_/Abs_control_ − Abs_blank_) × 100

where Abs_sample_ was the absorbance of the extract sample (diluted sample + DPPH solution), Abs_blank_ was the absorbance of the blank for each sample dilution (diluted sample + methanol) and Abs_control_ was the absorbance of control (sample solvent (methanol or water) + DPPH solution).

The value obtained for each concentration was plotted to obtain IC_50_ values (concentration in which the 50% of the free radical DPPH is reduced) in each time point.

### 2.5. 2,2-Azinobis-(3-Ethylbenzothiazoline-6-Sulfonate) (ABTS^•+^) Inhibition

In a 96-well microplate, 18 μL of sample were mixed with the ABTS^•+^ solution (ABTS^•+^ tab + K_2_S_2_O_8_ 140 mM + H_2_O) and, after 6 min, the absorbance was read at 734 nm. To build the calibration curve, Trolox (T) was used as standard compound (concentration range: 0–1000 µM) and the results were expressed as mM T equivalents (TE)/100 g d.w. phenolic extract [23].

### 2.6. Oxygen Radical Absorbance Capacity (ORAC)

The antiradical activity was further tested with the peroxyl radical 2,2′-azobis(2-amidinopropane) dihydrochloride (AAPH) by using the ORAC method [24]. Briefly, the sample was mixed with sodium fluorescein and fluorescence was measured at T0 using a microplate reader (excitation wavelength of 485 nm and emission wavelength of 520 nm). After adding the AAPH solution, measurements were carried out every 45 s for a total analysis time of 1 h. A standard curve was built by plotting T concentrations (4–250 μM) against the average net areas under the curve (AUC) of 3 measurements for each concentration. Final ORAC values were calculated using the regression equation between T concentrations and the net AUC and were expressed as mM TE/100 g d.w. phenolic extract.

### 2.7. High-Performance Liquid Chromatography (HPLC)

Individual polyphenols were determined by using a reversed-phase HPLC (Waters 2695, Milford, MA, USA), which was equipped with a C18 column (Nova-Pak, 150 × 3.9 mm, 4 μm, Waters), a 600 E multi-solvent delivery system, a Waters U6K sampler and a Waters 2996 photodiode array detector (DAD) [22]. Briefly, a gradient elution of polyphenols was performed using a binary solvent system (acetonitrile and acidified distilled water (pH 2 with orthophosphoric acid)) under isothermal conditions (25 °C). A detection wavelength range of 210–550 nm was used. The identification of the single compounds was confirmed by comparing their retention times and UV-DAD spectra with those of the corresponding standards, as well as with those reported in the literature [25]. The amount of each compound (expressed as area unit (AU) × 10^6^), was calculated before and after digestion.

### 2.8. Statistical Analysis

All experiments were run in triplicate. The results were expressed as mean values and standard deviation (SD). To test the normal distribution of data (*p* < 0.05) and the homogeneity of variance (*p* < 0.05), the Shapiro–Wilk method and the Levene and Bartlett tests were applied, respectively. A one-way analysis of variance (ANOVA), followed by Tukey’s honest significance test at a 95% confidence level (*p* ≤ 0.05), were performed to separate statistically different means between the non-digested (ND) and digested samples. To evaluate the correlation between data, Pearson’s correlation analysis (*p* < 0.05) was performed. Data were analyzed using XL-STAT (7.5.2 version, Addinsoft, Paris, France). For DPPH^•^, IC_50_ values were obtained by GraphPad Prism v6.01 (GraphPad Software, La Jolla, CA, USA).

## 3. Results and Discussion

### 3.1. Phenolic Content of the Extracts before and after In Vitro Digestion

TP content of the three extracts obtained from OMWW were measured before (ND) and after *in vitro* digestion (OD, GD and ID).

**Figure 1:**
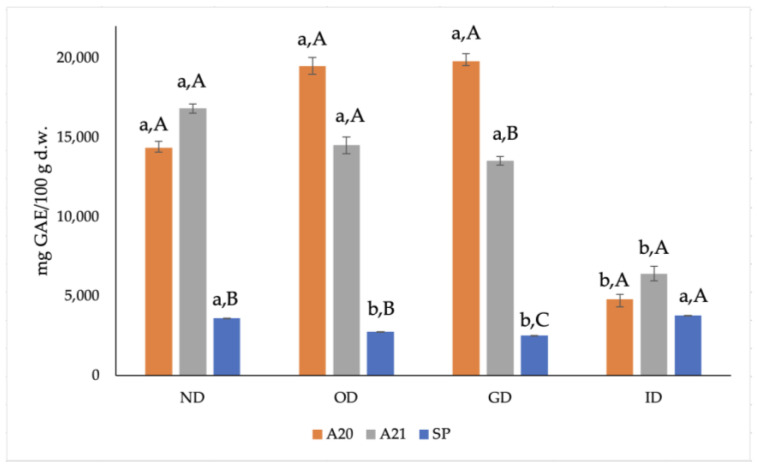
Effect of *in vitro* digestion on the phenolic content (expressed as mg GAE/100 g d.w.) of the three phenolic extracts. ND: non-digested; OD: oral digestion; GD: gastric digestion; ID: intestinal digestion; SP, spray-dried. Values are means of triplicates ± standard deviation (SD); a,b, statistically different means during digestion (*p* ≤ 0.05); A–C, statistically different means among samples (*p* ≤ 0.05).

In the present study, the two phenolic extracts were obtained by three-step membrane filtration and organic solvent extraction (A20 and A21), while the other extract (SP) was subjected to subsequent spray-drying. Figure 1 shows that both samples A20 and A21 had a significantly higher (*p* < 0.05) TP content than the SP sample, before and after *in vitro* digestion.

As can be noted from Figure 1, the TP content of the A20 and A21 samples was seven times higher than that of the SP sample before *in vitro* digestion, which was probably due to the preparation process of the extracts. In fact, A20 and A21 are purified viscous organic extracts, while SP is a powdered extract stabilized with maltodextrins that protect phenols. The TP values found in the present study were much higher (up to four times) than the one reported for an OMWW phenolic extract from Tunisian olives from cv. Chemlali [26]. This difference could be due to the extraction method used to obtain the phenolic extract. In fact, Ladhari et al. [26] performed a direct solvent extraction using methanol without a pre-concentration step, while in the present study a sequence of membrane filtration processes was carried out, followed by extraction with ethyl acetate.

During the *in vitro* digestion, it can be noted that the TP content of the A20 and A21 samples significantly varied, while it remained almost constant for the SP sample, with a significant, slight decrease during OD and GD. A20 and A21 displayed non-significant increasing and decreasing trends, respectively, in the OD and GD; such changes could be attributed to the action of the digestive enzymes as well as to the alkaline pH during the *in vitro* digestion process. After the entire *in vitro* digestion (ID), the TP content of both A20 and A21 samples showed a statistically significant three-fold decrease with respect to the corresponding ND samples. This overall trend was probably due to the preparation process of the extracts; in fact, A20 and A21 are purified viscous organic extracts, while SP is a powdered extract stabilized with maltodextrins that protects phenols during the digestion. The use of polymers such as lecithins and maltodextrins is a common practice to stabilize and protect bioactive compounds, such as phenols, to allow them to exert their antioxidant action in food and during digestion [27]. Thanks to maltodextrins, in fact, the SP sample could be stored under vacuum at room temperature, unlike the A20 and A21 extracts, which must be stored at −20 °C.

Taticchi et al. [28] tested the protective effect of a phenolic extract (PE) from OMWW, stabilized with maltodextrins in a 1:1 ratio, on carotenoids and other phytonutrients during a home-cooking procedure to prepare tomato sauce; the authors proved that, during the different cooking steps, 60.1% of total phenols were retained by the tomato sauce. The antioxidant activity exerted by PE during the preparation and cooking of the tomato sauce was able to preserve a significantly high content of carotenoids (α- and β-carotene and *E-* + *Z*-lycopene) in the final sauce.

In another study, Barbieri et al. [18] demonstrated that the same PE (OMWW extract + maltodextrins in a 1:1 ratio) was successfully retained during storage (>56%) and grilling (>20%) of raw and grilled beef hamburgers, leading to an improvement of their overall oxidative stability and sensory quality, with a positive effect especially on the intensity of the red color (raw product) during storage.

Miraglia et al. [29] also confirmed a good retention (69.3%) of the same OMWW PE in cooked pink shrimps, which helps maintaining the overall chemical and microbial quality of the seafood product during storage.

### 3.2. Phenolic Composition of the Extracts before and after In Vitro Digestion

Table 2 shows the phenolic compounds identified in the extracts by using HPLC-DAD. Five main phenolic compounds were identified: hydroxytyrosol (3,4-DHPEA), oleacein (3,4-DHPEA-EDA), oleocanthal (*p*-HPEA-EDA), tyrosol (*p*-HPEA) and verbascoside (VB).

The composition and concentration of phenolic compounds in virgin olive oil (VOO) and its by-products depend on several factors: pedo-climatic, agronomic (irrigation, fertilization, harvesting and ripeness) and technological (post-harvest storage and extraction system). In addition, the different olive cultivars provide distinctive qualitative-quantitative phenolic profiles to VOO and its by-products [16].

Table 2 reports that samples A20 and A21 had a higher total phenol content than the SP sample, especially before undergoing *in vitro* digestion. Like in the TP content determined by the Folin–Ciocolteau method, this was probably due to the different extract preparation (purified viscous organic extracts vs. powdered extract stabilized with maltodextrins).

After the *in vitro* digestion process, the phenolic content significantly decreased in all samples. In the SP sample, a 50% decrease of the phenolic content was observed, while a massive loss of phenols was found in samples A20 and A21 (89.3% and 76.9%, respectively). Although samples A20 and A21 had the highest content of phenols after *in vitro* digestion, maltodextrin encapsulation still proved to better protect the phenolic compounds in the SP sample. Regarding the content of the single phenols, the most abundant phenol in all extracts was 3,4-DHPEA-EDA, followed by 3,4-DHPEA. However, not all phenols were found in the extracts; in fact, *p*-HPEA-EDA was not detected in the SP sample.

On the other hand, it seems that the pH influenced the behavior of each phenolic compound during the various phases of digestion. For instance, in OD at pH 6.5, 3,4 DHPEA-EDA was not detected in the SP sample, nor were decreases detected in both samples A20 and A21, with a consequent increase of the 3,4 DHPEA. During GD, however, the pH drops to 2.5, leading to a decrease of 3,4 DHPEA and an increase of 3,4 DHPEA-EDA for the SP sample, while their content decreased in A21. In the course of ID, the pH rises to 6.5, which causes a decrease of 3,4 DHPEA-EDA and 3,4 DHPEA in all samples. A similar behavior was found for *p*-HPEA and *p*-HPEA-EDA in samples A20 and A21; in the SP sample, *p*-HPEA-EDA was not present, while *p*-HPEA values during digestion remained almost constant, with only a slight increase during the GD and ID phases. Regarding VB, in all analyzed samples it tended to decrease during digestion, until its complete disappearance in sample A20 during ID.

The biochemistry underlying the digestibility of phenolic compounds is quite complex; in fact, as evidenced by a recent review [30], more research is required to understand how the various phenolic classes work and are biotransformed in the human body, and how this biotransformation impacts their bioactivity and bioavailability. In addition to pH, it has been shown that the enzymes used during the various phases of the *in vitro* digestion also influence the behavior of the various molecules and their destruction/formation [16].

Other studies found that polyphenols, after digestion, increased the population of *Lactobacillus* spp., *Enterococcus* spp. and *Bifidobacterium* spp. while it decreased the population of bacteria that are occasionally linked to dysbiosis (i.e., *Clostridium histolyticum*, *Clostridium perfringens*, *Clostridium difficile* and others) [30]. These results highlight the potential of phenolic compounds as microbiota modulators and their application in the development of novel foods.

### 3.3. Antioxidant Activity of the Extracts before and after In Vitro Digestion

The antioxidant activity of SP, A20 and A21 extract was measured using three assays that deal with different radicals: ABTS^•+^, AAPH and DPPH^•^.

**Figure 2:**
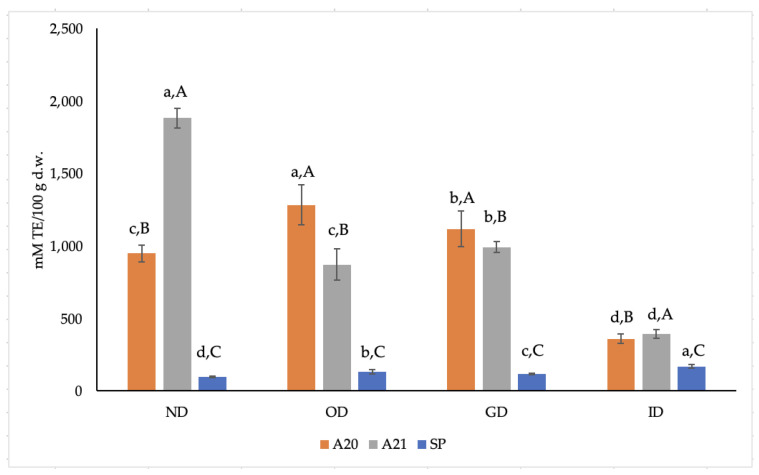
Effect of *in vitro* digestion on ABTS^•+^ inhibition (expressed as mM TE/100 g d.w.) of the three phenolic extracts. ND: non-digested; OD: oral digestion; GD: gastric digestion; ID: intestinal digestion; SP, spray-dried. Values are means of triplicates ± standard deviation (SD); a–d, statistically different means during digestion (*p* ≤ 0.05); A–C, statistically different means among samples (*p* ≤ 0.05).

Figure 2 shows the radical scavenging ability against ABTS^•+^ of the three extracts before and after the different steps of *in vitro* digestion. ABTS^•+^ values of samples A20 and A21 were 9.8 and 19.5 times higher, respectively, than those of the SP sample before *in vitro* digestion. The ABTS^•+^ values found in the present study are similar to those reported by Dauber et al. [31] for phenolic extracts obtained from OMWW of different cultivars (Arbequina and Coratina) by CO_2_ supercritical fluid extraction with ethanol as a co-solvent.

As shown in Figure 2, in the A20 and A21 samples, ABTS^•+^ value tends to decrease during *in vitro* digestion, reaching a final 2.63- and 4.79-fold decrease with respect to those found in the corresponding ND samples. An opposite trend, however, was found for the SP sample, as the ABTS^+•^ value at the end of the digestion process was 1.75 times higher than the one found for the ND sample. In this case, the protective role of maltodextrin towards phenols in SP samples is also evident, as is the effect of ID conditions on phenols’ release from the solid matrix.

It can be noted that the trends of ABTS^•+^ in the three extracts during *in vitro* digestion are similar to those found for TP (Figure 1), thus confirming the importance of phenols’ presence and content to detect their ABTS^•+^ scavenging activity [31].

**Figure 3:**
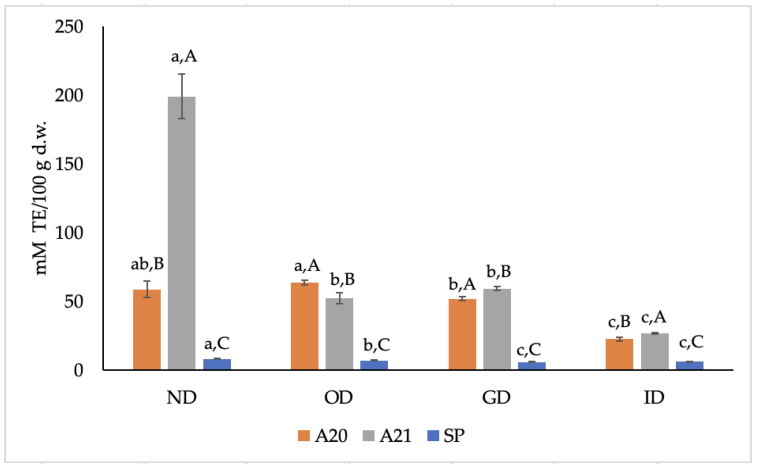
Effect of *in vitro* digestion on 2,2′-azobis(2-amidinopropane) dihydrochloride (AAPH) radical (expressed as mM TE/100 g d.w.) of the three phenolic extracts. ND: non-digested; OD: oral digestion; GD: gastric digestion; ID: intestinal digestion; SP, spray-dried. Values are means of triplicates ± standard deviation (SD); a–c, statistically different means during digestion (*p* ≤ 0.05); A–C, statistically different means among samples (*p* ≤ 0.05).

Figure 3 reports the radical scavenging ability against AAPH radical of the three extracts before and during the *in vitro* digestion, which showed the same trends as ABTS^+•^ (Figure 2). In brief, the ORAC values of A20 and A21 samples were 7.3 and 24.8 times higher, respectively, than those of the SP sample before *in vitro* digestion. Our ORAC values are comparable to those reported by Dauber et al. [31] for phenolic extracts produced from OMWW of different cultivars (Arbequina and Coratina) by using diverse extraction methods. After *in vitro* digestion, a 2.62- and 7.43-fold reduction of the ORAC value was found in samples A20 and A21 after ID, respectively, with respect to the corresponding ND samples; a 1.32-fold decrease of SP sample was also observed.

Regarding the DPPH test, values are expressed as IC_50_, which is the concentration of phenolic extract necessary to inhibit oxidation by 50%; this means that the more anti-radical capacity it has, the smaller the amount of extract is required to reduce oxidation by 50%.

As shown in Table 3, the value of IC_50_ in the A20 sample varied between 2.11 and 36.47 μg TE/mg during the *in vitro* digestion, while it ranged between 5.64 and 22.47 μg TE/mg in the A21 sample. In both extracts, IC_50_ tended to increase up to the GD phase, but they seem to lose their antioxidant capacity at the ID phase; this was more evident for A21.

On the other hand, the IC_50_ value of the SP sample varied from 147.62 to 49.87 μg TE/mg, with an increasing antioxidant capacity during *in vitro* digestion. In this case, a larger amount of ND extract is required to exert its antioxidant activity as compared to the one needed after *in vitro* digestion.

This behavior could be due to the protection provided to the phenolic extract by the encapsulation with maltodextrins; in fact, once the SP extract is subjected to *in vitro* digestion, the various enzymatic treatments at different pH conditions lead to the digestion of maltodextrins and the release of the phenolic fraction, the latter thus able to exert its antioxidant action even at a lower amount of SP sample. According to Burgos-Edwards et al. [32], the use of simulated digestion has a different impact on the antioxidant activity of phenolic extracts, which depends on the assay being used. In fact, the different methods used for the determination of phenols might not be able to reveal the structural changes in polyphenols and their associated activity [33]. Moreover, it should be pointed out that polyphenols and other antioxidant compounds could be metabolized by gut microbiota [34]. Consequently, all these results should be carefully interpreted and confirmed by future studies that involve intestinal fermentation in *in vitro* models to assess the metabolizing capacity of the gut microbiota, as well as the modulating effects on the microbiota itself [35].

### 3.4. Pearson’s Correlation Matrix

To evaluate the correlations between the different parameters analyzed, a Pearson correlation (*p* < 0.05) was performed. From the Pearson correlation matrix (Table 4), it is possible to observe how TP (content of phenols determined by the Folin–Ciocolteau assay) is positively correlated with some of the single phenols (*p*-HPEA, 3,4-DHPEA-EDA, *p*-HPEA-EDA), total phenols (determined by HPLC-DAD), ABTS^•+^ and AAPH; however, it is negatively correlated with the IC_50_ parameter. The latter negative correlation can be attributed to the fact that the types of extracts tested here display two different behaviors during the digestive process; in fact, as reported in Table 3, the quantities of extract necessary to detect antioxidant activity increase during digestion for samples A20 and A21, while they decrease for SP.

Regarding the single phenolic compounds, it is possible to note how *p*-HPEA is positively correlated with 3,4-DHPEA-EDA and *p*-HPEA-EDA, as well as with ABTS^+•^. It is known that *p*-HPEA-EDA is subjected to time-dependent hydrolysis under acid conditions in the stomach, which leads to an increase of free *p*-HPEA; in fact, after only 30 min of GD, higher amounts of *p*-HPEA may be present for absorption in the jejunum and ileum [36].

On the other hand, 3,4-DHPEA-EDA is positively correlated with 3,4-DHPEA, *p*-HPEA-EDA, ABTS^+•^ and AAPH. *p*-HPEA-EDA, instead, proved to be positively correlated with ABTS^+•^. The behavior and correlations of 3,4-DHPEA-EDA and 3,4-DHPEA can be ascribed to the fact that, following ingestion of 3,4-DHPEA-EDA-rich olive oil or olive by-product extracts, 3,4-DHPEA-EDA is hydrolyzed into 3,4-DHPEA and elenolic acid during digestion and further biotransformed [36]. In fact, according to the data in Table 2, 3,4-DHPEA-EDA is primarily hydrolyzed during gastrointestinal digestion, thus yielding 3,4-DHPEA, whose high antioxidant efficiency is attributed to its *o*-dihydroxyphenyl moiety [36].

Finally, it is possible to observe that, in addition to being positively correlated with all the single phenols, the total phenols (determined by HPLC-DAD) are also directly correlated with ABTS^•+^ and AAPH, thus confirming that it is the set of phenolic compounds, and not only the single compound, that possesses an antioxidant capacity. As observed for TP, the HPLC’s total phenols are negatively correlated with IC_50_.

## 4. Conclusions

This study demonstrated that OMWW could be a good source of phenolic compounds, from which phenol-rich extracts with good antioxidant capacity can be obtained. From the chemical characterization of extracts, it emerged that, before *in vitro* digestion, the phenol content of the A20 and A21 samples was higher than that of the SP sample. After the digestion process, a 50% decrease of the phenolic content in the SP sample was observed, while a massive loss of phenols was found in samples A20 and A21 (89.3% and 76.9%, respectively).

Concerning extracts’ antioxidant activity, ABTS^•+^ and ORAC values decreased during *in vitro* digestion, except for SP, where the ABTS^•+^ in the digested sample was 1.75 times higher than in the non-digested one. IC_50_ increased during digestion of the A20 and A21 samples, evincing a loss of antioxidant capacity after the intestinal phase; an opposite IC_50_ trend was noted in SP, confirming the protective role of maltodextrins.

Finally, this work showed that, despite the severe loss of phenols, samples A20 and A21 still had the highest content of phenols after undergoing *in vitro* digestion. The spray-dried formulation with maltodextrins (SP) proved, in any case, to better protect the phenolic compounds, managing to maintain its antioxidant capacity even after being digested, thus representing a promising OMWW phenolic extract to be used in the food field. Both types of OMWW phenolic extracts depict valid alternatives for the formulation of clean label products, which could be helpful for further valorizing this olive processing by-product and encouraging circularity in the olive oil industry. However, considering the role of gut microbiota on bioactives’ biotransformation and the possible modulating effect of phenols on microbiota, it will be important in the future to evaluate the interaction between these phenol-rich by-product extracts and the microbiota, in order to unravel the interaction mechanisms between them and their impact on phenols’ bioactivity and bioavailability.

## Figures and Tables

**Table 1:** Composition of the simulating digestive fluids.

	Enzymes	Concentration	Solvent	Added Quantity
**Oral phase**	α-amylase	1.3 mg/mL	CaCl_2_ 1 mM	125 μL
**Gastric phase**	Pepsin	160 mg/mL	HCl 0.1 M	165 μL
**Intestinal phase**	Pancreatin solution: bile extract (1:1, *v*/*v*)	Pancreatin solution (4 mg/mL); bile extract (25 mg/mL)	NaHCO_3_ 0.1 M	1250 μL

**Table 2:** Effect of *in vitro* on the phenolic content (expressed as AU × 10^6^) of 3 phenolic extracts.

		3,4-DHPEA	*p*-HPEA	3,4-DHPEA-EDA	*p*-HPEA-EDA	VB	Total Phenols
**A20**	ND	12.56 ± 0.38 ^b,B^	3.81 ± 0.21 ^b,A^	72.40 ± 0.48 ^a,A^	9.50 ± 0.19 ^a,A^	2.52 ± 0.06 ^a,A^	100.81 ± 7.35 ^a,A^
	OD	17.89 ± 0.83 ^a,B^	7.32 ± 0.40 ^a,A^	46.35 ± 0.47 ^b,A^	7.25 ± 0.20 ^b,A^	1.39 ± 0.22 ^c,A^	80.22 ± 11.10 ^b,A^
	GD	5.69 ± 0.26 ^c,B^	2.47 ± 0.39 ^c,A^	49.20 ± 0.57 ^b,A^	6.97 ± 0.24 ^c,A^	1.95 ± 0.05 ^b,A^	66.31 ± 8.18 ^c,A^
	ID	5.50 ± 0.13 ^c,C^	*n.d. ^d,C^*	3.89 ± 0.07 ^c,B^	*n.d. ^d,B^*	*n.d. ^d,C^*	9.39 ± 1.59 ^d,B^
**A21**	ND	14.76 ± 1.78 ^b,A^	2.16 ± 0.07 ^a,B^	53.15 ± 5.64 ^a,B^	3.58 ± 0.11 ^a,B^	0.63 ± 0.03 ^a,C^	74.29 ± 7.76 ^a,B^
	OD	31.10 ± 1.15 ^a,A^	2.41 ± 0.12 ^a,B^	33.60 ± 1.36 ^b,B^	2.30 ± 0.13 ^b,B^	0.50 ± 0.01 ^b,C^	69.64 ± 7.68 ^b,B^
	GD	8.87 ± 0.41 ^d,A^	0.88 ± 0.02 ^b,B^	12.32 ± 1.11 ^c,B^	1.04 ± 0.02 ^c,B^	0.37 ± 0.01 ^c,C^	23.50 ± 1.48 ^c,B^
	ID	9.15 ± 0.16 ^c,A^	0.96 ± 0.02 ^b,A^	6.22 ± 0.74 ^d,A^	0.73 ± 0.01 ^d,A^	0.05 ± 0.00 ^d,B^	17.14 ± 1.92 ^d,A^
**SP**	ND	6.64 ± 0.67 ^c,C^	0.20 ± 0.01 ^c,C^	10.77 ± 0.06 ^a,C^	*n.d. ^C^*	1.10 ± 28.99 ^a,B^	18.37 ± 6.84 ^a,C^
	OD	7.18 ± 0.50 ^b,C^	0.21 ± 0.06 ^c,C^	*n.d. ^c,C^*	*n.d. ^C^*	0.91 ± 15.69 ^b,B^	8.31 ± 0.51 ^d,C^
	GD	5.18 ± 0.01 ^d,B^	0.30 ± 0.01 ^b,C^	3.93 ± 0.01 ^b,C^	*n.d. ^C^*	0.82 ± 12.19 ^c,B^	10.25 ± 0.97 ^b,C^
	ID	8.03 ± 0.07 ^a,B^	0.40 ± 0.07 ^a,B^	*n.d. ^c,C^*	*n.d. ^B^*	0.78 ± 14.88 ^d,A^	9.22 ± 0.70 ^c,B^

3,4-DHPEA, hydroxytyrosol; 3,4-DHPEA-EDA, oleacein; *p*-HPEA, tyrosol; *p*-HPEA-EDA, oleocanthal; GD, gastric digestion; ID, intestinal digestion; ND, non-digested; *n.d*., not detected; OD, oral digestion; SP, spray-dried; VB, verbascoside. Values are means of triplicates ± standard deviation (SD). ^a–d^, statistically different means during digestion (*p* ≤ 0.05); ^A–C^, statistically different means among samples at each digestion step (*p* ≤ 0.05).

**Table 3:** Effect of *in vitro* digestion on 2,2-diphenyl-1-picrylhydrazyl (DPPH^•^) inhibition of the three phenolic extracts, calculated as IC_50_.

		15	30	45	60	75	90
		Min					
**A20**	ND	2.64 ± 0.21 ^c,C^	2.14 ± 0.16 ^d,C^	2.41 ± 0.23 ^c,C^	2.25 ± 0.23 ^d,C^	2.18 ± 0.23 ^c,C^	2.11 ± 0.22 ^c,C^
	OD	17.75 ± 1.64 ^b,C^	17.67 ± 2.14 ^c,B^	19.14 ± 2.95 ^b,B^	17.09 ± 2.95 ^c,B^	15.41 ± 1.55 ^b,B^	15.17 ± 1.03 ^b,B^
	GD	22.47 ± 0.87 ^a,C^	21.47 ± 0.80 ^b,B^	21.27 ± 1.20 ^b,B^	21.33 ± 1.20 ^b,B^	21.23 ± 1.41 ^a,B^	21.40 ± 1.43 ^a,B^
	ID	0 ^d,B^	36.74 ± 0.00 ^a,B^	31.52 ± 1.70 ^a,B^	27.81 ± 1.70 ^a,B^	0 ^d,B^	0 ^d,B^
**A21**	ND	7.36 ± 0.54 ^b,B^	6.91 ± 0.40 ^c,B^	6.56 ± 0.29 ^c,B^	6.21 ± 0.26 ^c,B^	5.93 ± 0.28 ^c,B^	5.64 ± 0.29 ^c,B^
	OD	25.35 ± 2.14 ^a,B^	15.53 ± 1.73 ^b,B^	15.47 ± 2.09 ^b,C^	14.17 ± 1.29 ^bC,^	14.43 ± 1.65 ^b,C^	13.70 ± 0.96 ^b,C^
	GD	22.47 ± 1.57 ^a,C^	21.47 ± 0.85 ^a,B^	21.27 ± 1.27 ^a,B^	21.33 ± 1.25 ^a,B^	21.23 ± 1.26 ^a,B^	21.40 ± 1.38 ^a,B^
	ID	0 ^c,B^	0 ^d,C^	0 ^d,C^	0 ^d,C^	0 ^d,B^	0 ^d,B^
**SP**	ND	169.08 ± 20.31 ^a,A^	156.06 ± 15.91 ^a,A^	151.85 ± 13.08 ^a,A^	147.62 ± 10.71 ^a,A^	159.11 ± 10.29 ^a,A^	159.59 ± 12.05 ^a,A^
	OD	102.24 ± 14.99 ^c,A^	96.90 ± 15.85 ^c,A^	95.73 ± 16.74 ^c,A^	94.48 ± 16.89 ^c,A^	96.66 ± 16.95 ^c,A^	99.39 ± 15.70 ^c,A^
	GD	143.29 ± 10.12 ^b,A^	131.03 ± 8.24 ^b,A^	128.86 ± 7.87 ^b,A^	131.68 ± 7.35 ^b,A^	133.94 ± 7.71 ^b,A^	134.77 ± 7.06 ^b,A^
	ID	53.36 ± 1.69 ^d,A^	49.48 ± 1.70 ^d,A^	49.87 ± 2.01 ^d,A^	52.52 ± 2.11 ^d,A^	53.99 ± 2.78 ^d,A^	75.34 ± 12.14 ^d,A^

GD, gastric digestion; ID, intestinal digestion; ND, non-digested; OD, oral digestion; SP, spray-dried. Values are means of triplicates ± standard deviation (SD); ^a–d^, statistically different means during digestion (*p* ≤ 0.05); ^A–C^, statistically different means among samples (*p* ≤ 0.05).

**Table 4:** Pearson’s correlation matrix among total and single phenols and antioxidant activity of the extracts before and after *in vitro* digestion.

	TP	3,4-DHPEA	*p*-HPEA	3,4-DHPEA-EDA	*p*-HPEA-EDA	VB	Total Phenols	ABTS^•+^	AAPH	IC_50_ 15 Min	IC_50_ 30 Min	IC_50_ 45 Min	IC_50_ 60 Min	IC_50_ 75 Min	IC_50_ 90 Min
TP	-														
3,4-DHPEA	0.503	-													
*p*-HPEA	0.777 *	0.521	-												
3,4-DHPEA-EDA	0.843 *	0.437 *	0.745 *	-											
*p*-HPEA-EDA	0.809 *	0.294	0.827 *	0.935 *	-										
VB	0.433	−0.019	0.525	0.703 *	0.808 *	-									
Total Phenols	0.863 *	0.595 *	0.813 *	0.980 *	0.915 *	0.645 *	-								
ABTS^•+^	0.910 *	0.462	0.645 *	0.797 *	0.663 *	0.243	0.794 *	-							
AAPH	0.666 *	0.370	0.380	0.645 *	0.413	0.051	0.618 *	0.910 *	-						
IC_50_ 15 min	−0.613 *	−0.344	−0.427	−0.476	−0.481	0.040	−0.497	−0.624 *	−0.485	-					
IC_50_ 30 min	−0.685 *	−0.436	−0.492	−0.546	−0.539	−0.029	−0.580 *	−0.680 *	−0.528	0.979 *	-				
IC_50_ 45 min	−0.678 *	−0.429	−0.479	−0.540	−0.531	−0.016	−0.572	−0.676 *	−0.528	0.984 *	1.000*	-			
IC_50_ 60 min	−0.682 *	−0.435	−0.485	−0.544	−0.533	−0.013	−0.576 *	−0.679 *	−0.530	0.986 *	0.998 *	0.999 *	-		
IC_50_ 75 min	−0.627 *	−0.387	−0.438	−0.488	−0.484	0.046	−0.516	−0.633 *	−0.492	0.999 *	0.981 *	0.986 *	0.989 *	-	
IC_50_ 90 min	−0.654 *	−0.400	−0.457	−0.517	−0.505	0.039	−0.544	−0.660 *	−0.514	0.990 *	0.972 *	0.977 *	0.982 *	0.994 *	-

* indicates significant correlation between parameters (*p* < 0.05). AAPH, 2,2’-azobis(2-amidinopropane) dihydrochloride; 3,4-DHPEA, hydroxytyrosol; 3,4-DHPEA-EDA, oleacein; *p*-HPEA, tyrosol; *p*-HPEA-EDA, oleocanthal; TP, total polyphenol content; VB, verbascoside.

## Data Availability

Not applicable.
